# Greenhouse gas emissions and carbon sequestration by agroforestry systems in southeastern Brazil

**DOI:** 10.1038/s41598-017-16821-4

**Published:** 2017-12-01

**Authors:** Carlos Moreira Miquelino Eleto Torres, Laércio Antônio Gonçalves Jacovine, Sílvio Nolasco de Olivera Neto, Clyde William Fraisse, Carlos Pedro Boechat Soares, Fernando de Castro Neto, Lino Roberto Ferreira, José Cola Zanuncio, Pedro Guilherme Lemes

**Affiliations:** 1 Departamento de Engenharia Florestal, Universidade Federal de Viçosa, 36570-900 Viçosa, Minas Gerais Brazil; 2 Department of Agricultural and Biological Engineering, University of Florida, P.O. Box 110570, Gainesville, FL32611 USA; 3 TTG Brasil Investimentos Florestais Ltda, 18470-130 Itapeva, São Paulo Brazil; 4 Departamento de Fitotecnia, Universidade Federal de Viçosa, 36570-900 Viçosa, Minas Gerais Brazil; 5 Departamento de Entomologia/BIOAGRO, Universidade Federal de Viçosa, 36570-900 Viçosa, Minas Gerais Brazil; 6 Instituto de Ciências Agrárias, Universidade Federal de Minas Gerais, 39404-547 Montes Claros, Minas Gerais Brazil

## Abstract

Agrosilvopastoral and silvopastoral systems can increase carbon sequestration, offset greenhouse gas (GHG) emissions and reduce the carbon footprint generated by animal production. The objective of this study was to estimate GHG emissions, the tree and grass aboveground biomass production and carbon storage in different agrosilvopastoral and silvopastoral systems in southeastern Brazil. The number of trees required to offset these emissions were also estimated. The GHG emissions were calculated based on pre-farm (e.g. agrochemical production, storage, and transportation), and on-farm activities (e.g. fertilization and machinery operation). Aboveground tree grass biomass and carbon storage in all systems was estimated with allometric equations. GHG emissions from the agroforestry systems ranged from 2.81 to 7.98 t CO_2_e ha^−1^. Carbon storage in the aboveground trees and grass biomass were 54.6, 11.4, 25.7 and 5.9 t C ha^−1^, and 3.3, 3.6, 3.8 and 3.3 t C ha^−1^ for systems 1, 2, 3 and 4, respectively. The number of trees necessary to offset the emissions ranged from 17 to 44 trees ha^−1^, which was lower than the total planted in the systems. Agroforestry systems sequester CO_2_ from the atmosphere and can help the GHG emission-reduction policy of the Brazilian government.

## Introduction

The Paris Agreement, adopted in the 21st session of the Conference of the Parties (COP 21) for the United Nations Framework Convention on Climate Change (UNFCCC), aims to maintain the global average temperature below 2 °C of pre-industrial levels^1^. The signatory countries stipulate their Intended Nationally Determined Contributions (INDCs), which are the main commitments and contributions of that country for the fulfillment of the agreement^2,3^.

The Brazilian INDC proposed to reduce the greenhouse gases (GHG) emission by 37% in 2025, based on 2005 levels^4^. Agriculture is the main emission source with enteric fermentation being responsible for 90% of CH_4_ and animal manure on pasture for 33% of N_2_O emissions in Brazil in 2014^5^. The Brazilian government established a “low-carbon agriculture plan” to promote sustainable practices in agriculture by reducing greenhouse gas (GHG) emissions while maintaining profitability^6^.

This plan is based on practices such as restoration of degraded pastures, crop-livestock-forest integration, no-till farming, biological nitrogen fixation and forestry and agroforestry systems^6^. The agroforestry system is a land use management system combining trees and/or woody perennial plants, pasture and livestock benefiting from ecological and economic interactions between its component parts due to production diversification^7^. Food production^8^ and carbon sequestration by tree planting^9^ in these systems can help to reduce deforestation in tropical countries^10,11^.

Agrosilvopastoral and silvopastoral systems are agroforestry system types that can reduce and offset GHG emissions from the Brazilian agricultural sector, mainly using cattle and forest integration^12–14^. These systems lower animal emission levels^12^ by improving grass quality, which can reduce CH_4_ emissions from enteric fermentation^15^ and digestion efficiency^16^. Furthermore, these systems may mitigate GHG emissions by enhancing carbon sequestration through increasing above and belowground biomass^17–19^.

The objective of this study was to estimate GHG emissions, tree and grass aboveground biomass and carbon storage in silvopastoral and agrosilvopastoral systems in southeastern Brazil, and the number of trees required to offset these emissions.

## Results

### GHG emissions

**Figure 1:**
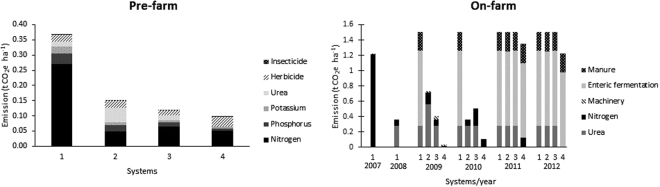
Greenhouse gas emissions (t CO_2_e ha^−1^) during pre-farm and on-farm stages in system 1, 2, 3 and 4.

The pre-farm GHG emissions were 0.37, 0.15, 0.12 and 0.10 t CO2e ha-1 in systems 1, 2, 3 and 4, respectively. Nitrogen production was the main emission source for pre-farm activities (Fig. 1). On-farm GHG emissions were 7.61, 4.10, 3.92 and 2.71 t CO_2_e ha^−1^ in systems 1, 2, 3 and 4, respectively. Enteric fermentation and manure produced by livestock were the main emission sources for on-farm activity (Fig. 1). Total GHG emissions were 7.98, 4.25, 4.04 and 2.80 t CO_2_e ha^−1^, on systems 1, 2, 3 and 4, respectively (Table 1).

**Table 1:** GHG emission, carbon stock, carbon balance, trees to offset, total trees, surplus trees in all systems.

System	Emission (t CO_2_e ha^−1^)	Carbon Stock (t CO_2_e ha^−1^)	Balance (t CO_2_e ha^−1^)	Trees to offset	Total trees	Surplus Trees
1	7.98	200.14	192.16	17	424	407
2	4.25	41.86	37.61	39	388	349
3	4.04	94.33	90.29	44	1031	987
4	2.81	21.78	18.97	35	267	232

### Aboveground biomass and carbon storage

The equation m1 was the best to predict aboveground biomass and carbon storage in systems 1, 3 and 4 (Tables 2 and 3). These equations had the highest *R* ^2^_*adj*_, and lower RMSE (%) than the m2. In system 2, the equation m1 was rejected due to the incoherence of the values for parameter b21, with negative values (Tables 2 and 3).

**Table 2:** Estimated regression coefficients and adjusted standard errors (±SE) coefficient of determination **(** *R* ^2^_*adj*_ **)**, model bias ($\bar{E}$), and root mean square error (±RMSE) of aboveground biomass equations.

System	Model	Parameter	Estimate	SE	*R* ^*2*^_*adj*_(%)	$\bar{E}$ (%)	RMSE (%)
1	m_1_	b_01_	0.0204	0.0371	85.409	−0.549	19.588
		b_11_	1.9908	0.6453			
		b_21_	1.0264	0.8239			
	m_2_	b_02_	0.0208	0.0313	85.407	−0.551	19.589
		b_12_	1.0035	0.1581			
2	m_1_	b_01_	0.1563	0.1784	90.699	−0.221	16.911
		b_11_	3.0839	1.0899			
		b_21_	−0.7883	1.2929			
	m_2_	b_02_	0.0601	0.0586	88.996	0.040	18.390
		b_12_	0.8691	0.1163			
3	m_1_	b_01_	0.0737	0.1087	90.182	−0.922	19.953
		b_11_	1.8942	0.5883			
		b_21_	0.6112	0.9028			
	m_2_	b_02_	0.0534	0.0480	90.112	−0.857	20.023
		b_12_	0.8678	0.1086			
4	m_1_	b_01_	0.0895	0.0659	94.741	−0.769	16.781
		b_11_	1.4018	0.6370			
		b_21_	1.0429	0.8270			
	B_2_	b_02_	0.1027	0.0543	94.706	−0.857	16.836
		b_12_	0.7960	0.0621

**Table 3:** Estimated regression coefficients and adjusted standard errors (±SE), adjusted coefficient of determination (*R* ^*2*^_*adj*_), model bias ($\bar{E}$), and root mean square error (±RMSE) of carbon equations.

System	Model	Parameter	Estimate	SE	*R* ^*2*^_*adj*_(%)	$\bar{E}$ (%)	RMSE (%)
1	m_1_	b_01_	0.0108	0.0197	85.439	−0.541	19.550
		b_11_	1.9977	0.6440			
		b_21_	1.0153	0.8218			
	m_2_	b_02_	0.0110	0.0165	85.439	−0.542	19.550
		b_12_	1.0031	0.1578			
2	m_1_	b_01_	0.0842	0.0960	90.663	−0.226	16.906
		b_11_	3.0889	1.0900			
		b_21_	−0.8036	1.2927			
	m_2_	b_02_	0.0322	0.0314	88.930	0.039	18.409
		b_12_	0.8663	0.1162			
3	m_1_	b_01_	0.0382	0.0564	90.193	−0.920	19.920
		b_11_	1.8823	0.5867			
		b_21_	0.6251	0.9013			
	m_2_	b_02_	0.0282	0.0253	90.131	−0.859	19.982
		b_12_	0.8666	0.1083			
4	m_1_	b_01_	0.0475	0.0344	94.868	−0.736	16.536
		b_11_	1.3937	0.6278			
		b_21_	1.0498	0.8151			
	m_2_	b_02_	0.0547	0.0285	94.831	−0.827	16.596
		b_12_	0.7950	0.0612			

The tree stems were responsible for 90, 70, 76 and 70% of the total aboveground biomass on systems 1, 2, 3 and 4, respectively. The branches were responsible for 4, 18, 14 and 15% and the leaves for 6, 12, 10 and 15% of the total aboveground biomass in systems 1, 2, 3 and 4, respectively. The leaves had the highest carbon content in all systems (57.0, 55.2, 56.0 and 56%), followed by the stem (52.4, 52.1, 52.2 and 52.3%), and the branches (52.1, 51.3, 50.5 and 52.3%). Carbon storage in trees and grass aboveground biomass was 54.58, 11.42, 25.73, 5.94 t C ha^-1^ and 3.28, 3.60, 3.77, 3.32 t C ha^−1^, for systems 1, 2, 3 and 4, respectively.

A total of 17, 39, 44 and 35 trees ha^−1^ are necessary to offset all GHG emissions, which is equivalent to 4.0, 10.2, 3.4 and 13.1% of the total numbers of trees in systems 1, 2, 3 and 4, respectively (Table 1).

## Discussion

The average annual GHG emissions ranged from 0.93 to 1.60 t CO_2_e ha^−1^ yr^−1^, which may be considered low when compared to other systems^20,21^, probably due to the use of no-till farming and the adoption of agroforestry systems with reduced machinery use, fuel inputs and CO_2_ emissions^22^. No-till farming in these systems may increase organic carbon and nitrogen content in the soil, and the microbial biomass, mitigating GHG emissions^23–26^. Usual management practices in agroforestry systems, such as no-till farming and optimal fertilization/manure regimes can increase carbon sequestration while reducing GHG emissions^27^. Such a combination provides additional environmental benefits such as soil erosion reduction and prevention^28,29^, more efficient water-use^30^, and improvement in biodiversity^31^.

The difference in the mean annual aboveground carbon increment (MAI-AGB) on the four systems indicates that the amount of this element sequestered may depend on tree species, age, geographic location, environmental factors, and system management^32,33^. System 1 presented the largest MAI-AGB (11.19 t ha^−1^ yr^−1^) due to its older age and the fertilization carried out to enhance maize production indirectly increasing tree biomass^34^. System 3 presented the second largest IMA due to its greater plant density (9 × 1 spacing), however competition between plants can negatively affect individual growth^35,36^ and may increase future mortality^37^. All systems were important in carbon sequestration and had environmental benefits such as soil fertility and water quality improvement and erosion reduction^18,38–40^. The estimated MAI-AGB found was higher than the 1.43 t ha^−1^ yr^−1^ in a silvopastoral system with 105 trees per hectare (eucalypt and acacia) in Minas Gerais, Brazil^41^. The MAI-AGB of 7.67 t ha^−1^ yr^−1^ of an agrosilvopastoral system with eucalyptus spaced 10 × 4 m and rice in Paracatu, Minas Gerais, Brazil^42^ was similar to that observed in the system 2 of this research.

The estimated aboveground grass carbon sequestration was similar to the 3.71 kg C ha^−1^ of an agrosilvopastoral system with eucalypt in Minas Gerais, Brazil^42^, and the 3.29 kg C ha^−1^ of a silvopastoral system with 200 pine trees ha^−1^ in São Paulo, Brazil^43^. These systems had a similar production due to the wide spacing of the trees, allowing sufficient radiation transmittance^18^ and improving the microclimate for the forage^15,44^. This shows that agroforestry systems are an alternative to recover degraded pasture land by improving chemical, physical and biological soil conditions and enhancing carbon sequestration^12,18,45–47^.

The number of trees required to offset GHG emissions was lower than that planted in the systems studied, demonstrating their great potential to sequester carbon and to reduce GHG emissions.^12,48,49^. Agroforestry systems are important for the “Low-Carbon Agriculture Plan” of the Brazilian government to achieve GHG emission-reduction targets. These systems decrease the pressure on forests^48^, and improve animal welfare and crop production^12^. Furthermore, the remaining sequestered carbon can be sold in voluntary markets with a higher price for technologies that bring social and environmental benefits including higher farmer income^50^.

The systems had a positive carbon balance and a tree surplus ranging from 232 to 987. The number of trees was higher than necessary to offset GHG emissions in all systems. Therefore, the agroforestry systems can effectively mitigate GHG emissions.

## Methods

### Study systems

The study was conducted in silvopastoral and agrosilvopastoral systems in Viçosa, Minas Gerais, Brazil. The climate in this region is humid subtropical with dry winters and hot summers, classified as Cwa (Köppen classification). The average annual temperature and rainfall are 19.4 °C and 1,200 mm, respectively. The soil is classified as red-yellow latosol and the topography ranges from strongly undulated to mountainous with an average altitude of 689.7 m.

The agrosilvopastoral systems were composed of maize (*Zeya mays*) and *Eucalyptus saligna* (system 1), and bean (*Phaseolus vulgaris*) and *E. urophylla* x *E. grandis* (system 2) during the first year, and the crops were replaced by pasture (*Brachiaria decumbens*) with livestock grazing in the second year (Table 4). The silvopastoral systems (3 and 4) had pasture (*Brachiaria decumbens*) + *E. urophylla x E. grandis* (Table 4). No-till farming was used in all systems. Beef cattle were reared in all systems (one animal/ha).

**Table 4:** Study area characterization.

System	Crop	Pasture	Planting	Area (ha)	Tree arrangements (m)
1	Maize	Brachiaria	Dec/2007	0.93	8 × 3
2	Beans	Brachiaria	Dec/2009	0.72	8 × 3
3	—	Brachiaria	Dec/2009	0.55	9 × 1
4	—	Brachiaria	Nov/2009	3.48	12 × 3

System 1 was fertilized after soil analysis. In December 2007, a posthole digger machine was used and 0.2 kg of N-P-K (06-30-06) applied per tree hole. Additional fertilization of 0.16 kg of N-P-K (20-05-20) pit^−1^ was carried out three months after tree planting. Weeds and leaf-cutting ants were controlled before, during and after tree planting. Animal traction was used to apply 500 kg of N-P-K (08-24-12) ha^−1^ on maize before planting, and another 500 kg of N-P-K (30-00-10) ha^−1^ 30 days later. The pasture received 100 kg of urea ha^−1^ year^−1^.

Systems 2 and 3, implemented in December 2009, received the same treatment as the system 1 for eucalypt planting and weed/leaf-cutter ant control. In system 2, the bean crop received 250 kg of N-P-K (08-28-16) ha^−1^ and 200 kg urea ha^−1^ as top-dressing fertilization.

The eucalypt trees in system 4 were planted, in November 2009, using a posthole digger machine with 0.2 kg N-P-K (06-30-06) applied per hole. Additional fertilization with 0.05 kg N-P-K (20-05-20) plant^−1^, 0.1 kg of N-P-K (20-05-20) plant^−1^, 0.15 kg and 0.1 kg KCl plant^−1^ were undertaken at 60, 120, 300 and 550 days after tree planting, respectively. Weed and leafcutter ants were controlled before, during and after tree planting. The pasture received 100 kg of N-P-K (20-05-20) ha^−1^ one year after eucalypt planting.

### GHG emissions

**Figure 2:**
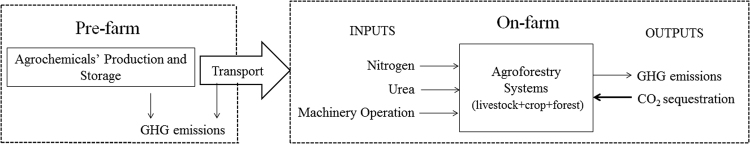
GHG inventory for the agroforestry systems, for pre-farm and on-farm stages.

GHG emission calculations per system were based on pre-farm activities, such as production, storage, and transportation of agrochemicals, and on-farm activities such as fertilization and machinery use (Fig. 2). The data were estimated from personal interviews with farmers. They were asked to report on the use of machine fuel, agrochemicals and estimated crop yield.

Pre-farm emissions were calculated using emission factors (Table 5)^51^ and the following equation: emAgr = agrochemical *EF*(44/12), EmAgr = annual emissions resulting from production, packaging, storage and distribution of agrochemicals, kg CO_2_ year^−1^; agrochemical = agrochemical applied, kg year^−1^; EF = emission factor, kg carbon equivalent kg^−1^; 44/12 = C to CO_2_ conversion factor.

**Table 5:** Carbon emissions (mean ± SD) for the production, transportation, storage, and transfer of agrochemicals. Values according to a previous study^51^.

Agrochemicals	Carbon emission (kg C kg substance^−1^)
Nitrogen fertilizer	1.30 ± 0.30
Phosphorus fertilizer	0.20 ± 0.06
Potassium fertilizer	0.15 ± 0.06
Urea	0.16 ± 0.11
Herbicide	6.30 ± 2.70
Insecticide	5.10 ± 3.00
Fungicide	3.90 ± 2.20

On-farm emissions were calculated based on the “Guidelines for National Greenhouse Gas Inventories”^52^. GHG sources included nitrogen fertilization, farm machinery, enteric fermentation, and manure management.

Input emissions from synthetic fertilizers were calculated via two pathways: direct and indirect. The direct emissions refer to mineral fertilizer applications^52^. Direct emissions are the product of the nitrogen applied by the emission factor (0.01)^52^ using the 44/28 factor to convert N_2_ to N_2_O, and N_2_O global warming potential (298 units of CO_2_e)^53^. The equation used to estimate direct emissions was: Em_DiF_ = F_SN/FRP_*EF_1_*(44/28)*GWP; Em_DiF_ = direct CO_2_e emissions from N inputs to managed soils, kg CO_2_ ha^−1^; F_SN_ = annual amount of synthetic fertilizer N applied to soils, kg N ha^−1^; F_PRP_ = annual amount of dung and urine N deposited on soils, kg N^−1^; EF_1_ = emission factor developed for N_2_O emissions from synthetic fertilizer, kg N_2_O–N (kg N)^−1^; 44/28 = N_2_ to N_2_O conversion factor; GWP = global warming potential.

Indirect emissions result from volatilization, atmospheric deposition of NH_3_ and NOx, and nitrogen leaching and runoff from the fertilizers^54,55^. Indirect emissions were calculated using annual amount of fertilizer N applied to soils and the nitrogen fraction lost by volatilization, leaching and/or runoff^56^. The emission factor was 0.01 for volatilization and 0.0075 for leaching/runoff. The nitrogen fraction lost due to volatilization and leaching/runoff was fixed as 0.1 and 0.2, respectively^52^. The equation used to estimate indirect on-farm N_2_O emissions per system was Em_LnL_ = F_SN_*Frac_LEACH-(H)_*EF_3_*(44/28)*GWP, where Em_LnL_ = amount of CO_2_e produced from additions to managed soils, kg CO_2_ ha^−1^; F_SN_ = amount of synthetic fertilizer N applied to soils, kg N ha^−1^; EF_3_ = emission factor for N_2_O emissions from N leaching and runoff, kg N_2_O–N (kg N leached and runoff)^−1^; Frac_LEACH-(H)_ = fraction of all N added to/mineralized in managed soils in regions where leaching/runoff occurs that is lost through leaching and runoff, kg N (kg of N additions)^−1^; GWP = global warming potential.

NO_2_ emissions from urea were calculated with the same equations used for the other nitrogen fertilizers. CO_2_ emissions were the product of the urea applied to the soil by its emission factor, 0.20^52^. The equation used to estimate on-farm CO_2_ emissions was Em_Urea_ = M*EF_4,_ where Em_Urea_ = amount of CO_2_e produced from urea application, t CO_2_ ha^−1^; M = amount of urea applied to soils, t N ha^−1^; EF_4_ = emission factor for applied urea, t of C (ton of urea)^−1^.

CO_2_ emissions from agricultural machinery were those generated by fuel consumption during eucalypt planting due to its emission factor (EF_5_), 2.327 kg CO_2_^−1^ ^52^. The equation used in each system was Em_D_ = F*EF_5_, where Em_D_ = amount of CO_2_e produced from fuel consumed, kg CO_2_ ha^−1^; F = fuel consumed, L ha^−1^; EF_5_ = emission factor, kg C (L fuel)^−1^.

The CH_4_ emissions by enteric fermentation from cattle were calculated using the factor of 39 kg CH_4_ year^−1^ animal unit^−1^ ^57^. The equation used was: Em_FE_ = N* EF_6_* GWP, where Em_FE_ = emissions from enteric fermentation, kg CO_2_ ha^−1^; N = number of animals, head ha^−1^; EF_6_ = emission factor for enteric fermentation (kg CH_4_) head^−1^; GWP = CH_4_ global warming potential. N_2_O emissions due to manure deposition were calculated with the same equations as those for nitrogen fertilizer.

### Carbon storage in aboveground biomass

Ten pasture grass samples (1 m^2^) between tree rows were collected, per season, from June 2012 to October 2013. Their fresh weight was obtained and the fresh:dry weight ratio calculated with 25 g from each sample. These samples were dried at approximately 65 °C in an oven until weight stabilization.

The diameter at breast height (DBH), total height, and commercial height (stem height up to 3-cm diameter) of trees per system were measured between July and August 2012. Trees were grouped into DBH classes, and three individuals per class were selected and felled to determine their total volume, biomass and carbon levels in their stem, branches and leaves.

The trees selected were cut at ground level, and the stem diameters measured at 0.3, 0.7, and 1.3 m from their base, and thereafter at every 2 m until the diameter reached 3 cm. The volume of these stem sections was calculated using the Smalian’s formula^58^. The stems per sample were weighed and 2.5 cm thick stem discs were collected at the base, 25, 50, 75, and 100% of the commercial height to calculate the aboveground biomass. An additional stem disc was cut at breast height (1.3 m). The branches and stem discs were dried at 103 ± 2 °C until dry weight stabilization was reached. The leaf and branch weights per tree sampled were recorded. Fresh leaf and branch samples were weighed in the field, stored in bags and sent to the laboratory to determine their dry/fresh weight ratio^59^. Leaf and branch samples were dried at 65 ± 2 °C until dry weight stabilization.

The stem, leaf and branch carbon content was determined with a LECO TruSpec Micro CHN analyzer (LECO Corp., St. Joseph, MI). The carbon stock was obtained by multiplying the aboveground biomass by the carbon content.

Field data was fitted to allometric equations^60,61^ to estimate the tree aboveground biomass, and carbon (stem + branches + leaves) per system as: Y_1_ = β_01_*DBH^β11^*H ^β21^*ε_1_; Y_2_ = β_02_*(DBH^2^*H) ^β12^*ε_2_, whereYj the biomass or carbon stock (kg) of the j^th^ model; H total height (m); β_0i_, β_1i_, and β_2i_ the parameters of the j^th^ model and ε_i_:the random errors.

All statistical analyses were performed with R statistical software^62^. The best equations were based on the criteria: parameter significance (p < 0.05) by Wald test; coherence of the sign associated with a specific parameter; goodness of fit statistics: R^2^_adj_ = 1 − [(n − p − 1)/(n − p)] * (1 − R^2^); R^2^ = 1 − [Σ(y − $\hat{y}$)^2^/Σ(y − $\bar{y}$)^2^); RMES% = (100/$\overline{y}$) * $\sqrt{{\rm{\Sigma }}(y-\hat{y})2/n}$; $\bar{E} \%$ = (100/$\bar{y}$) * (Σ(y − $\hat{y}$)/n), where, R^2^ is the empirical determination coefficient or model efficiency; *R* ^2^_*adj*_, an empirical adjusted determination coefficient; $\bar{E}$%, a relative bias; RMSE%, the root square error in percentage; n, the observation number; p, the number of explanatory variables; $\bar{y}$, the mean of dependent variable (volume, biomass and carbon); y_i_, the i^th^ observed value; and $\hat{y}$, the i^th^ value of the dependent variable.
